# Average quantum dynamics of closed systems over stochastic Hamiltonians

**DOI:** 10.1038/s41598-025-14825-z

**Published:** 2025-08-23

**Authors:** Li Yu, Daniel F. V. James

**Affiliations:** 1 Department of Physics, University of Toronto, 60 St. George Street, Toronto, ON M5S 1A7 Canada; 2 Department of Physics, Harvard University, Cambridge, MA 02138 USA

**Keywords:** Quantum physics, Physics, Atomic and molecular physics

## Abstract

We develop a formally exact master equation to describe the evolution of the average density matrix of a closed quantum system driven by a stochastic Hamiltonian. The average over stochastic processes generally results in decoherence effects in closed system dynamics, in addition to the unitary evolution. We then show that, for an important class of problems in which the Hamiltonian is proportional to a Gaussian random process, the 2nd-order master equation yields exact dynamics. The general formalism is applied to study the examples of a two-level system, two atoms in a stochastic magnetic field and the heating of a trapped ion, where we find phenomena such as decoherence-induced disentanglement.

## Introduction

The density operator encapsulates all the statistical information about the state of a quantum system. The evolution of the density operator of a closed system is governed by the Hamiltonian. In practice, the Hamiltonian can seldom be strictly determined or precisely controlled – it fluctuates both in a temporal sense and between repeated realizations, which can be mathematically described by random processes. Therefore, instead of treating any Hamiltonian as deterministic in an idealized manner, we would like to take such fluctuations into account explicitly when studying quantum dynamics. Our goal is to obtain the *average* dynamics in the following sense: Suppose an ensemble of systems are prepared in some initial state and subsequently evolve under a randomly fluctuating Hamiltonian, how does the density matrix that describes the ensemble as a whole evolve?

Note that we would like to consider closed, but not isolated, systems with Hamiltonians determined by some classical stochastic quantities. Such classical stochastic quantities can arise from the environment, e.g. an external magnetic field that fluctuates across different realizations of an experiment. Such random fluctuations arise when experimentalists do not have precise control over the physical quantities of the environment. A closed but not isolated system can exchange energy with the environment, but does not get entangled with the environment, so that its evolution in each realization of the experiment is unitary. We are treating the environment classically, not as quantum degrees of freedom. In this paper, we would like to study how such classical environments affect the dynamics of quantum systems through stochastic Hamiltonians.

Previous work on stochastic average dynamics was done by Budini^1^ using a variational calculus method and Novikov’s theorem, and by Guha et al.^2^ using a non-perturbative cluster cumulant method. Kiely^3^ has worked on exact master equations for a number of specific noise types. There is a study by Groszkowski et al.^4^ on driven systems subject to classical non-Markovian noise using a method based on generalized cumulant expansions. There is also a study on qubit dynamics with classical noise^5^ through an approach other than master equation. Kropf et al.^6^ derived general evolution equations for the ensemble-average quantum dynamics generated by disordered Hamiltonians. There is also a study by Cai et al.^7^ on the decoherence of quantum systems in stochastically fluctuating environments. A different kind of average dynamics over the time domain was studied by Gamel and James^8^, assuming the deterministic (i.e. non-stochastic) Hamiltonian but taking into account the finite time-window of measurements. The master equation formalism is also widely used in the study of open systems dynamics^9^. It should be noted that, despite the formal similarity, our study is on the dynamics of *closed* quantum systems and no environment is involved. One of the authors has also worked on quantum dynamics under the joint scenario of quantum and classical noises^10^.

In this paper, we will first adopt a series expansion approach and derive a time-local master equation that describes the ensemble-average dynamics of a general quantum system. The general formalism is then used to study a representative class of Hamiltonians obeying Gaussian statistics. Finally, we apply the master equation method to some physical examples and find interesting phenomena such as fluctuation-induced decoherence and decoherence-induced disentanglement. Throughout, our results are compared to exact dynamics and the validity of the master equation approach is discussed.

## Theory

### Ensemble-average density matrix

Consider a closed, but not isolated, system for which the Hamiltonian is determined by some classical stochastic quantity *x*(*t*). Suppose an experiment is carried out repeatedly with each realization labelled by $\mu$. The evolution of the density matrix $\rho ^{\mu }(t)$ that describes the quantum system in the $\mu$-th realization is governed by the Hamiltonian $\hat{H}^{\mu }(t)=\hat{H}[x^{\mu }(t)]$, and is given by

1 $$\begin{aligned} \rho ^{\mu }(t)=\hat{U}^{\mu }(t,t_{0})\rho _{0}\hat{U}^{\mu \dagger }(t,t_{0}), \end{aligned}$$

where $\rho _{0}$ is the initial density matrix, which is assumed to be uncorrelated with *x*(*t*) and thus is the same in all realizations. The unitary evolution operator $\hat{U}^{\mu }(t,t_{0})$ obeys the equation of motion,

2 $$\begin{aligned} i\hbar \frac{\partial }{\partial t}\hat{U}^{\mu }(t,t_{0})=\hat{H}^{\mu }(t)\hat{U}^{\mu }(t,t_{0}). \end{aligned}$$

The average density matrix $\overline{\rho }(t)$ is defined as follows,

3 $$\begin{aligned} \overline{\rho }(t)\equiv \underset{N\rightarrow \infty }{\lim }\frac{1}{N}\overset{N}{\underset{\mu =1}{\sum }}\rho ^{\mu }(t). \end{aligned}$$

It can be shown that $\overline{\rho }(t)$ is Hermitian, positive and of unit trace, which is ensured by the properties of the individual density matrices $\rho ^{\mu }(t)$. Thus the operator $\overline{\rho }(t)$ is indeed a physical density matrix, describing the average statistics of the ensemble of realizations as a whole.

The equation of motion for $\overline{\rho }(t)$ is formally given by

4 $$\begin{aligned} i\hbar \frac{\partial }{\partial t}\overline{\rho }(t)=\underset{N\rightarrow \infty }{\lim }\frac{1}{N}\overset{N}{\underset{\mu }{\sum }}i\hbar \frac{\partial }{\partial t}\rho ^{\mu }(t)=\underset{N\rightarrow \infty }{\lim }\frac{1}{N}\overset{N}{\underset{\mu }{\sum }}[\hat{H}^{\mu }(t),\rho ^{\mu }(t)]=\overline{[\hat{H}(t),\rho (t)]}. \end{aligned}$$

However, since the right hand side cannot be written as a function of $\overline{\rho }(t)$, the equation is not of a closed form and thus not very useful. With the goal of obtaining a closed equation for $\overline{\rho }(t)$, we resort to a series expansion approach.

### Series expansion of the evolution operator

Following the standard recipe for perturbative expansion^11^, the unitary operator $\hat{U}^{\mu }(t,t_{0})$ in a particular realization $\mu$ can be written as a power series in $\lambda$ (a parameter controlling the “strength” of the Hamiltonian):

5 $$\begin{aligned} \hat{U}^{\mu }(t,t_{0})=\overset{\infty }{\underset{n=0}{\sum }}\lambda ^{n}\hat{U}_{n}^{\mu }(t,t_{0}) \end{aligned}$$

where

6 $$\begin{aligned} \hat{U}_{0}^{\mu }(t,t_{0})= & \hat{I}, \end{aligned}$$

7 $$\begin{aligned} \hat{U}_{n}^{\mu }(t,t_{0})= & \frac{1}{i\hbar }\intop _{t_{0}}^{t}dt'\hat{H}^{\mu }(t')\hat{U}_{n-1}^{\mu }(t',t_{0}),\, n\geqslant 1. \end{aligned}$$

Thus $\overline{\rho }(t)$ can be expressed in terms of $\hat{U}_{n}^{\mu }(t,t_{0})$ and $\lambda$:

8 $$\begin{aligned} \overline{\rho }(t)=\overline{\left( \underset{m}{\sum }\lambda ^{m}\hat{U}_{m}(t,t_{0})\right) \rho _{0}\left( \underset{n}{\sum }\lambda ^{n}\hat{U}_{n}^{\dagger }(t,t_{0})\right) }=\overset{\infty }{\underset{k=0}{\sum }}\lambda ^{k}{\mathscr {E}}_{k}[\rho _{0}]\equiv {\mathscr {E}}[\rho _{0}], \end{aligned}$$

where ${\mathscr {E}}_{k}[\rho _{0}]$ is the time-dependent map defined as

9 $$\begin{aligned} {\mathscr {E}}_{k}[\rho _{0}]\equiv \overset{k}{\underset{j=0}{\sum }}\overline{\hat{U}_{k-j}(t,t_{0})\rho _{0}\hat{U}_{j}^{\dagger }(t,t_{0})}, \end{aligned}$$

and ${\mathscr {E}}[\rho _{0}]$ is a completely positive linear map^12^. Although their argument is a density matrix in this instance, ${\mathscr {E}}_{k}$ and ${\mathscr {E}}$ can act on any operator in general.

### Inverse transformation

The map ${\mathscr {E}}$ is a linear transformation that maps $\rho _{0}$ to $\overline{\rho }$. Since both $\rho _{0}$ and $\overline{\rho }$ are operators in the same Hilbert space, and thus of the same dimension, it is natural to postulate that an inverse transformation ${\mathscr {F}}={\mathscr {E}}^{-1}$ exists that maps $\overline{\rho }$ to $\rho _{0}$. That is,

10 $$\begin{aligned} \rho _{0}={\mathscr {E}}^{-1}[\overline{\rho }]\equiv {\mathscr {F}}[\overline{\rho }]. \end{aligned}$$

Such an inverse should exist in the weak coupling or short time regimes^9^. Note that the meaning of “inverse” is purely mathematical here: the map ${\mathscr {E}}^{-1}$ is not to be confused with an inverse dynamical evolution in the physical sense. According to the semigroup property, a completely positive, trace preserving (CPTP) linear map is physically invertible if and only if it is an unitary map (see Section 3.8 of Ref.^13^). Therefore, in general, a CPTP map ${\mathscr {E}}:\,\rho _{0}\rightarrow \overline{\rho }$ does not have a physical inverse, that is, we cannot find another CPTP map that gives $\overline{\rho }\rightarrow \rho _{0}$. However, the mathematical inverse ${\mathscr {E}}^{-1}$ that serves our purpose here needs not be CPTP.

Since the composition of a transformation and its inverse is the identity transformation, the identity ${\mathscr {F}}[{\mathscr {E}}[\rho ]]={\mathscr {I}}[\rho ]$ holds for an arbitrary operator $\rho$. Following^8^, we adopt the ansatz that ${\mathscr {F}}$ can be expanded in powers of $\lambda$, ${\mathscr {F}}[\rho ]=\overset{\infty }{\underset{m=0}{\sum }}\lambda ^{m}{\mathscr {F}}_{m}[\rho ]$. Then we have

11 $$\begin{aligned} \overset{\infty }{\underset{m=0}{\sum }}\lambda ^{m}{\mathscr {F}}_{m}[\overset{\infty }{\underset{n=0}{\sum }}\lambda ^{n}{\mathscr {E}}_{n}[\rho ]]=\overset{\infty }{\underset{k=0}{\sum }}\lambda ^{k}\left( \overset{k}{\underset{j=0}{\sum }}{\mathscr {F}}_{j}[{\mathscr {E}}_{k-j}[\rho ]]\right) =\lambda ^{0}{\mathscr {I}}[\rho ]. \end{aligned}$$

Collecting terms of like powers in $\lambda$, we obtain the set of equations involving ${\mathscr {F}}_{m}$ and ${\mathscr {E}}_{n}$:

12 $$\begin{aligned} {\mathscr {F}}_{0}[{\mathscr {E}}_{0}[\rho ]]= & {\mathscr {I}}[\rho ], \end{aligned}$$

13 $$\begin{aligned} {\mathscr {F}}_{0}[{\mathscr {E}}_{1}[\rho ]]+ & {\mathscr {F}}_{1}[{\mathscr {E}}_{0}[\rho ]]=0,\end{aligned}$$

14 $$\begin{aligned} {\mathscr {F}}_{0}[{\mathscr {E}}_{2}[\rho ]]+ & {\mathscr {F}}_{1}[{\mathscr {E}}_{1}[\rho ]]+{\mathscr {F}}_{2}[{\mathscr {E}}_{0}[\rho ]]=0, \end{aligned}$$

and so on. Solving for ${\mathscr {F}}_{m}$ in terms of ${\mathscr {E}}_{n}$, and making use of ${\mathscr {E}}_{0}={\mathscr {I}}$ as defined in Eq. (9), we have

15 $$\begin{aligned} {\mathscr {F}}_{0}[\rho ]= & {\mathscr {E}}_{0}[\rho ]={\mathscr {I}}[\rho ],\end{aligned}$$

16 $$\begin{aligned} {\mathscr {F}}_{1}[\rho ]= & -{\mathscr {E}}_{1}[\rho ],\end{aligned}$$

17 $$\begin{aligned} {\mathscr {F}}_{2}[\rho ]= & -{\mathscr {E}}_{2}[\rho ]+{\mathscr {E}}_{1}[{\mathscr {E}}_{1}[\rho ]], \end{aligned}$$

and so on. We could have worked out higher-order terms ${\mathscr {F}}_{m}[\rho ]$ for $m\geqslant 3$, which would have paved the way for a formally exact master equation in the subsequent section.

### Master equation

Differentiating Eq. (8) with respect to time and making use of the inverse relation in Eq. (10), we obtain the following equation:

18 $$\begin{aligned} i\hbar \frac{\partial }{\partial t}\overline{\rho }(t)=i\hbar \dot{{\mathscr {E}}}[\rho _{0}]=i\hbar \dot{{\mathscr {E}}}[{\mathscr {F}}[\overline{\rho }(t)]]. \end{aligned}$$

Here, the notation $\dot{{\mathscr {E}}}[\rho ]$ means first taking time-derivative of the time-dependent transformation ${\mathscr {E}}$ to obtain a new transformation denoted by $\dot{{\mathscr {E}}}$ and then letting $\dot{{\mathscr {E}}}$ act on $\rho$; the argument $\rho$ is not differentiated whether or not it is time-dependent. Assuming the order of differentiation and summation can be switched, we have

19 $$\begin{aligned} \dot{{\mathscr {E}}}[{\mathscr {F}}[\overline{\rho }(t)]]=\overset{\infty }{\underset{n=0}{\sum }}\lambda ^{n}\dot{{\mathscr {E}}_{n}}[\overset{\infty }{\underset{m=0}{\sum }}\lambda ^{m}{\mathscr {F}}_{m}[\overline{\rho }(t)]]=\overset{\infty }{\underset{k=0}{\sum }}\lambda ^{k}\left( \overset{k}{\underset{j=0}{\sum }}\dot{{\mathscr {E}}_{j}}[{\mathscr {F}}_{k-j}[\overline{\rho }(t)]]\right) ; \end{aligned}$$

thus the equation of motion can be written as

20 $$\begin{aligned} i\hbar \frac{\partial }{\partial t}\overline{\rho }(t)=\overset{\infty }{\underset{k=0}{\sum }}\lambda ^{k}\left( i\hbar \overset{k}{\underset{j=0}{\sum }}\dot{{\mathscr {E}}_{j}}[{\mathscr {F}}_{k-j}[\overline{\rho }(t)]]\right) \equiv \overset{\infty }{\underset{k=0}{\sum }}\lambda ^{k}{\mathscr {L}}_{k}[\overline{\rho }(t)]. \end{aligned}$$

Evaluating ${\mathscr {F}}_{m}$ and $\dot{{\mathscr {E}}_{n}}$ explicitly, we find

21 $$\begin{aligned} {\mathscr {L}}_{0}[\rho ]= & i\hbar \dot{{\mathscr {E}}_{0}}[{\mathscr {F}}_{0}[\rho ]]=0,\end{aligned}$$

22 $$\begin{aligned} {\mathscr {L}}_{1}[\rho ]= & i\hbar \dot{{\mathscr {E}}_{0}}[{\mathscr {F}}_{1}[\rho ]]+i\hbar \dot{{\mathscr {E}}_{1}}[{\mathscr {F}}_{0}[\rho ]]=\overline{\hat{H}}\rho -\rho \overline{\hat{H}},\end{aligned}$$

23 $$\begin{aligned} {\mathscr {L}}_{2}[\rho ]= & i\hbar \dot{{\mathscr {E}}_{0}}[{\mathscr {F}}_{2}[\rho ]]+i\hbar \dot{{\mathscr {E}}_{1}}[{\mathscr {F}}_{1}[\rho ]]+i\hbar \dot{{\mathscr {E}}_{2}}[{\mathscr {F}}_{0}[\rho ]]\nonumber \\= & \overline{\hat{H}\hat{U_{1}}}\rho -\overline{\hat{H}}\,\overline{\hat{U}_{1}}\rho +\overline{\hat{H}\rho \hat{U}_{1}^{\dagger }}-\overline{\hat{H}}\rho \overline{\hat{U}_{1}^{\dagger }}\nonumber \\ & -\rho \overline{\hat{U}_{1}^{\dagger }\hat{H}}+\rho \overline{\hat{U}_{1}^{\dagger }}\,\overline{\hat{H}}-\overline{\hat{U}_{1}\rho \hat{H}}+\overline{\hat{U}_{1}}\rho \overline{\hat{H}}, \end{aligned}$$

and so on. Here we could have systematically worked out the higher-order terms ${\mathscr {L}}_{k}[\rho ]$ for $k\geqslant 3$, which would result in a formally exact master equation. Note again that the argument $\rho$ is not to be averaged or differentiated and that terms like $\overline{\hat{H}}$ are time-dependent just as ${\mathscr {L}}_{k}[\rho ]$ are time-dependent transformations.

Keeping terms up to 2nd order and setting $\lambda =1$ in Eq. (20), a time-local master equation is thus obtained for the evolution of $\overline{\rho }(t)$:

24 $$\begin{aligned} i\hbar \frac{\partial }{\partial t}\overline{\rho }(t)=[\overline{\hat{H}},\overline{\rho }(t)]+\hat{A}\overline{\rho }(t)-\overline{\rho }(t)\hat{A}^{\dagger }+{\mathscr {D}}[\overline{\rho }(t)], \end{aligned}$$

where $\hat{A}\equiv \overline{\hat{H}\hat{U}_{1}}-\overline{\hat{H}}\,\overline{\hat{U}_{1}}$ and ${\mathscr {D}}[\rho ]\equiv \overline{\hat{H}\rho \hat{U}_{1}^{\dagger }}-\overline{\hat{H}}\rho \overline{\hat{U}_{1}^{\dagger }}-\overline{\hat{U}_{1}\rho \hat{H}}+\overline{\hat{U}_{1}}\rho \overline{\hat{H}}$. The effective Hamiltonian responsible for unitary evolution is

25 $$\begin{aligned} \hat{H}_{eff}\equiv \overline{\hat{H}}+\frac{1}{2}(\hat{A}+\hat{A}^{\dagger }), \end{aligned}$$

with which the master equation can be written in a more insightful way,

26 $$\begin{aligned} i\hbar \frac{\partial }{\partial t}\overline{\rho }(t)=[\hat{H}_{eff},\overline{\rho }(t)]+\frac{1}{2}\left\{ \hat{A}-\hat{A}^{\dagger },\overline{\rho }(t)\right\} +{\mathscr {D}}[\overline{\rho }(t)]. \end{aligned}$$

This result is formally similar to a previous work on average dynamics^8^. However, the physical meaning is different since the derivation in that case is for a time-average density matrix in a *single* realization. Although both averaging approaches lead to non-unitary dynamics, the physical origins differ significantly. The time averaging arises from our inability to resolve fast dynamics within a measurement window, with the time evolution being coarse-grained, whereas the ensemble averaging in this case arises from the variability of repeated realizations of an experiment, with each experimental run following a stochastically different Hamiltonian trajectory. Incidentally, our result may also be reminiscent of some master equations for the reduced density matrix of open quantum systems. But it should be emphasized that our derivation is for a closed system and thus quantum entanglement with environment does not play a role here.

Note that the above results are formally applicable to an interaction-picture density matrix, though we implicitly assume the Schrodinger picture in the derivation. The only difference is in the interpretation of the density matrix: When we use the interaction-picture density matrix $\overline{\rho }_{I}$, the expectation value of an observable $\hat{O}$ is given by $\langle \hat{O}\rangle =Tr\left( \hat{O}_{I}\overline{\rho }_{I}\right)$, where $\hat{O}_{I}$ is the interaction-picture operator instead of the original operator in Schrodinger picture.

In the above, we have worked out the ensemble average dynamics up to second order, as this is the order where leading-order decoherence effects will occur. Besides the commutator with the effective Hamiltonian, the rest of the terms on the right-hand side of Eq. (26) describe such decoherence effects. Decoherence in the case of ensemble average closed system dynamics are seemingly similar to decoherence in open system dynamics. Despite this apparent similarity, they are conceptually different. In the former case, the off-diagonal elements of the density matrix (say in some “pointer basis”) rotate at stochastically different rates across different realizations of the stochastic process, so when we take the ensemble average, the off-diagonal elements across different realizations tend to cancel out, resulting in vanishing off-diagonal elements. Roughly speaking, this is how decoherence arises in ensemble average closed systems. (Decoherence in the ensemble average sense often goes by the name of “dephasing” in the literature.) In the case of open system dynamics, we talk about a single realization of an experiment only, and there is no such kind of ensemble average to be taken. In this case, however, the system gets physically entangled with the environment. And an off-diagonal element of the system’s reduced density matrix is equal to the overlapping integral of the respective environmental parts of the total wavefunction. Because of the largeness of the environmental Hilbert space, this overlapping integral between the respective environmental parts of the wavefunction can quickly become vanishingly small, resulting in decoherence in open systems. We should pay attention to the conceptual difference between these two scenarios of decoherence despite their apparent similarity.

Also note that Eq. (26) is a non-Markovian master equation. And characteristic of such non-Markovianity is the possibility of negative decoherence rates^4^. This corresponds to possible revival of coherence in the ensemble average dynamics. Physically, this means quantum information could sometimes flow back into the system from the (classical) environment during the evolution. By contrast, decoherence rates remain positive at all times in Markovian dynamics, with quantum information being constantly lost to the environment in the evolution.

## General applications

### Time-independent Hamiltonian

Let us first apply our general result to the simple case where the parameters in the Hamiltonian are time-independent. That is, $\hat{H}=\hat{H}(a)$, where *a* represents random variable(s) instead of random process(es). Suppose further that $\hat{H}$ is of zero-mean, which implies a particular choice of “picture”: Any time-independent, deterministic part of the Hamiltonian plus the average component of the stochastic part can be removed by a gauge transformation, that is, by switching to an suitably chosen interaction picture^14^. Note that, in the case of time-independent random variables, $\hat{U}_{1}(t,t_{0})=(t-t_{0})\hat{H}/i\hbar$ and $\hat{U}_{1}^{\dagger }(t,t_{0})=-\hat{U}_{1}(t,t_{0})$, thus $[\hat{U}_{1},\,\hat{H}]=[\hat{U}_{1}^{\dagger },\,\hat{H}]=0$. So we have $\hat{A}+\hat{A}^{\dagger }=0$ and thus $\hat{H}_{eff}=0$. This result is special to the time-independent case, however. As we will see later, the effective Hamiltonian (to 2nd order) is in general non-zero, due to the non-commutativity of $\hat{U}_{1}$ and $\hat{H}$ in the time-dependent case. After simplification, the 2nd-order master equation in this particular case is

27 $$\begin{aligned} \frac{\partial }{\partial t}\overline{\rho }(t)=-\frac{t}{\hbar ^{2}}\{\overline{\hat{H^{2}}},\overline{\rho }(t)\}+\frac{2t}{\hbar ^{2}}\overline{\hat{H}\overline{\rho }(t)\hat{H}}. \end{aligned}$$

A class of problems of physical interest has a Hamiltonian of the form

28 $$\begin{aligned} \hat{H}=\hbar \underset{n}{\sum }a_{n}\hat{h}_{n}+a_{n}^{*}\hat{h}_{n}^{\dagger }, \end{aligned}$$

where $a_{n}$ are jointly circular complex Gaussian random variables of zero mean. Substituting Eq. (28) into Eq. (27), we find

29 $$\begin{aligned} \frac{\partial }{\partial t}\overline{\rho }(t)=t\underset{k,l}{\sum }\{\Gamma _{kl}\left( -\hat{h}_{k}\hat{h}_{l}^{\dagger }\overline{\rho }(t)-\overline{\rho }(t)\hat{h}_{k}\hat{h}_{l}^{\dagger }+2\hat{h}_{l}^{\dagger }\overline{\rho }(t)\hat{h}_{k}\right) +\Gamma _{kl}^{*}\left( -\hat{h}_{k}^{\dagger }\hat{h}_{l}\overline{\rho }(t)-\overline{\rho }(t)\hat{h}_{k}^{\dagger }\hat{h}_{l}+2\hat{h}_{l}\overline{\rho }(t)\hat{h}_{k}^{\dagger }\right) \}, \end{aligned}$$

where $\Gamma _{kl}=\overline{a_{k}a_{l}^{*}}$ are the correlation functions.

### Single real Gaussian random process

Now consider the case of a time-dependent Hamiltonian

30 $$\begin{aligned} \hat{H}(t)=\hbar a(t)\hat{h}, \end{aligned}$$

where *a*(*t*) is a (real) Gaussian random process. This is representative of a wide class of problems, for example, the Zeeman effect, where *a*(*t*) is proportional to the external magnetic field and $\hat{h}$ is the *z*-component of the total angular momentum^15^. The random process in this section is taken to be the most general case, that is, we do not assume any additional property like zero-mean or stationarity.

The ensemble-average dynamics under this Hamiltonian is exactly solvable, so let us first work out the exact, analytic result. The unitary evolution operator in a particular realization $\mu$ is

31 $$\begin{aligned} \hat{U}^{\mu }(t,t_{0})=\exp \left( -iv^{\mu }(t)\hat{h}\right) , \end{aligned}$$

where

32 $$\begin{aligned} v^{\mu }(t)\equiv \intop _{t_{0}}^{t}dt'a^{\mu }(t'). \end{aligned}$$

For an initial state $\rho (t_{0})=\underset{k,l}{\sum }\rho _{kl}(t_{0})|k\rangle \langle l|$, where $\left\{ |n\rangle \right\}$ is the energy-eigenbasis with $\hat{h}|n\rangle =E_{n}|n\rangle$, the evolution in a particular realization is

33 $$\begin{aligned} \rho ^{\mu }(t)=\hat{U}^{\mu }(t,t_{0})\rho (t_{0})\hat{U}^{\mu \dagger }(t,t_{0})=\underset{k,l}{\sum }\rho _{kl}(t_{0})\exp \left\{ -iv^{\mu }(t)(E_{k}-E_{l})\right\} |k\rangle \langle l|, \end{aligned}$$

thus the ensemble-average is

34 $$\begin{aligned} \overline{\rho }(t)=\underset{k,l}{\sum }\rho _{kl}(t_{0})\overline{\exp \left\{ -iv(t)(E_{k}-E_{l})\right\} }|k\rangle \langle l|. \end{aligned}$$

Since *v*(*t*) is a linear filtered Gaussian random process, it is a Gaussian random process itself. (See page 83 of Ref.^16^.) Invoking the special properties of Gaussian statistics, it can be shown that

35 $$\begin{aligned} \overline{\exp \left\{ -iv(t)(E_{k}-E_{l})\right\} }=\exp \left\{ -i(E_{k}-E_{l})\overline{v(t)}-\frac{(E_{k}-E_{l})^{2}}{2}\left[ \overline{v(t)^{2}}-\overline{v(t)}^{2}\right] \right\} . \end{aligned}$$

Thus, the exact ensemble-average dynamics is given by the elements of the average density matrix:

36 $$\begin{aligned} \overline{\rho }_{kk}(t)= & \rho _{kk}(t_{0}),\end{aligned}$$

37 $$\begin{aligned} \overline{\rho }_{kl}(t)= & \rho _{kl}(t_{0})\exp \left\{ -i(E_{k}-E_{l})\overline{v(t)}-\frac{(E_{k}-E_{l})^{2}}{2}[\overline{v(t)^{2}}-\overline{v(t)}^{2}]\right\} . \end{aligned}$$

Now let us solve the same problem by the master equation approach. Using the results from Eqs.(25, 26), the following expression is obtained,

38 $$\begin{aligned} \begin{aligned} i\hbar \frac{\partial }{\partial t}\overline{\rho }(t)=&[\hbar \overline{a(t)}\hat{h},\overline{\rho }(t)]+i\hbar \left[ \overline{a(t)}\intop _{t_{0}}^{t}dt'\overline{a(t')}-\intop _{t_{0}}^{t}dt'\overline{a(t)a(t')}\right] \left( \hat{h}^{2}\overline{\rho }(t)+\overline{\rho }(t)\hat{h}^{2}\right) \\&+2i\hbar \left[ \intop _{t_{0}}^{t}dt'\overline{a(t)a(t')}-\overline{a(t)}\intop _{t_{0}}^{t}dt'\overline{a(t')}\right] \hat{h}\overline{\rho }(t)\hat{h}, \end{aligned} \end{aligned}$$

which can be simplified to

39 $$\begin{aligned} \frac{\partial }{\partial t}\overline{\rho }(t)=-i\overline{a(t)}[\hat{h},\overline{\rho }(t)]+D(t)\left[ \hat{h},[\hat{h},\overline{\rho }(t)]\right] , \end{aligned}$$

where

40 $$\begin{aligned} D(t)\equiv \overline{a(t)}\intop _{t_{0}}^{t}dt'\overline{a(t')}-\intop _{t_{0}}^{t}dt'\overline{a(t)a(t')}. \end{aligned}$$

To find the solution to this differential equation, we first write it down in terms of the matrix elements in the $\hat{h}$-eigenbasis:

41 $$\begin{aligned} \frac{\partial }{\partial t}\overline{\rho }_{kk}(t)= & 0,\end{aligned}$$

42 $$\begin{aligned} \frac{\partial }{\partial t}\overline{\rho }_{kl}(t)= & \left[ -i\overline{a(t)}(E_{k}-E_{l})+D(t)(E_{k}-E_{l})^{2}\right] \overline{\rho }_{kl}(t),\,\,(k\ne l). \end{aligned}$$

Now we have a set of (de-coupled) linear ordinary differential equations (ODE’s)*,* which is easily solvable,

43 $$\begin{aligned} \overline{\rho }_{kk}(t)= & \rho _{kk}(t_{0}),\end{aligned}$$

44 $$\begin{aligned} \overline{\rho }_{kl}(t)= & \rho _{kl}(t_{0})\exp \left\{ \intop _{t_{0}}^{t}dt'\left[ -i\overline{a(t')}(E_{k}-E_{l})+D(t')(E_{k}-E_{l})^{2}\right] \right\} \nonumber \\= & \rho _{kl}(t_{0})\exp \left\{ -i(E_{k}-E_{l})\overline{v(t)}-\frac{(E_{k}-E_{l})^{2}}{2}[\overline{v(t)^{2}}-\overline{v(t)}^{2}]\right\} . \end{aligned}$$

The second equality in Eq. (44) is obtained after some calculation, where *v*(*t*) is given by Eq. (32). Thus we find the dynamics generated by the 2nd-order master equation *coincides* with the exact dynamics in this case.

We observe that the energy population is conserved during the evolution while the coherence between different energy levels decays. Thus the evolution of the average dynamics is pure decoherence, with the “pointer basis”^17^ being the energy-eigenbasis. Note that, although the Hamiltonian varies with time and across different realizations, the energy-eigenbasis is the same throughout. In the case where some energy level is degenerate, we readily have a “decoherence-free subspace”, in which quantum information can be stably stored^18^. Incidentally, a derivation in the context of open systems also suggests that energy eigenstates can emerge as “pointer states” in the so-called “quantum limit of decoherence”^19^. In that case, however, the decoherence results from small environmental perturbation to the system, not from the fluctuation of the system Hamiltonian itself.

We could have worked out the higher-order terms (i.e. ${\mathscr {L}}_{n}[\rho ]$ for $n\geqslant 3$) explicitly to see how accurate the 2nd-order approximation is. However, since the solution to the 2nd-order master equation coincides with the exact dynamics, we can readily conclude that all higher-order terms must sum up to zero without actually carrying out further calculations.

Note that when deriving Eq. (39) we do not assume anything about the nature of the random process *a*(*t*), not even the Gaussian statistics. In other words, the solution to the 2nd-order master equation is given by Eqs.(43, 44) in all cases. On the other hand, the exact dynamics Eqs.(36, 37) is based on the assumption of Gaussian statistics. If *a*(*t*) is *not* a Gaussian random process, then the exact dynamics will be different. The implication is that, for *a*(*t*) being non-Gaussian, the 2nd-order master equation is not exact.

### Multiple jointly circular complex Gaussian random processes

Let us briefly present the results for the more general Hamiltonian $\hat{H}(t)=\hbar \underset{n}{\sum }\left( a_{n}(t)\hat{h}_{n}+a_{n}^{*}(t)\hat{h}_{n}^{\dagger }\right)$, where $a_{n}(t)$ are jointly circular complex Gaussian random processes of zero mean. The 2nd-order master equation is found to be

45 $$\begin{aligned} \begin{aligned} \frac{\partial }{\partial t}\overline{\rho }(t)=&-\underset{k,l}{\sum }\alpha _{kl}(t)\left[ [\hat{h}_{k},\hat{h}_{l}^{\dagger }],\,\overline{\rho }(t)\right] +\underset{k,l}{\sum }\beta _{kl}(t)\\&\times \left\{ -\hat{h}_{k}\hat{h}_{l}^{\dagger }\overline{\rho }(t)-\overline{\rho }(t)\hat{h}_{k}\hat{h}_{l}^{\dagger }+2\hat{h}_{l}^{\dagger }\overline{\rho }(t)\hat{h}_{k}-\hat{h}_{l}^{\dagger }\hat{h}_{k}\overline{\rho }(t)-\overline{\rho }(t)\hat{h}_{l}^{\dagger }\hat{h}_{k}+2\hat{h}_{k}\overline{\rho }(t)\hat{h}_{l}^{\dagger }\right\} , \end{aligned} \end{aligned}$$

where

46 $$\begin{aligned} & \alpha _{kl}(t)\equiv \frac{1}{2}\intop _{t_{0}}^{t}dt'\left( \overline{a_{k}(t)a_{l}^{*}(t')}-\overline{a_{l}^{*}(t)a_{k}(t')}\right) , \end{aligned}$$

47 $$\begin{aligned} & \beta _{kl}(t)\equiv \frac{1}{2}\intop _{t_{0}}^{t}dt'\left( \overline{a_{k}(t)a_{l}^{*}(t')}+\overline{a_{l}^{*}(t)a_{k}(t')}\right) . \end{aligned}$$

By comparing with Eq. (29) for the time-independent Hamiltonian case, we notice a major difference in this case is that the effective Hamiltonian is non-zero despite $\overline{\hat{H}(t)}=0$. This effective unitary evolution results from the fact that the Hamiltonian operators at different times do not commute with each other in general.

The 2nd-order master equation yields exact dynamics only for the special case of a single real Gaussian random process. In this more general case, Eq. (45) does not lead to exact dynamics in general. This can be shown by explicitly evaluating higher-order terms like ${\mathscr {L}}_{4}[\rho ]$ to find that they do not vanish in general. Despite the lack of perfect agreement, the master equation is nevertheless of great use in such cases, because the exact dynamics is generally not obtainable and the 2nd-order master equation serves as a good approximation when the higher-order terms (e.g. ${\mathscr {L}}_{4}[\rho ]$) are small compared to ${\mathscr {L}}_{2}[\rho ]$.

## Physical examples

We will illustrate the general results by applying them to a few examples. The findings will then be used to gain physical insights, and the validity of the master equation approach will be examined by comparing to the exact dynamics.

### Two-level system

First consider a two-level system (e.g. spin-1/2) subject to the Hamiltonian $\hat{H}(t)=\hbar \omega (t)\hat{Z}$, where $\hat{Z}$ is the *z*-component of Pauli operator and $\omega (t)$ a stationary Gaussian random process of zero mean. (A two-level system subject to a stochastic Hamiltonian is characteristic of a wide range of physical phenomena, e.g. a spin-1/2 particle in a stochastic magnetic field. This model has been previously studied in^20,21^.) This falls into the category of Hamiltonians (30). Using Eq. (39), the 2nd-order master equation is obtained,

48 $$\begin{aligned} \frac{\partial }{\partial t}\overline{\rho }(t)=-\frac{1}{4}d(t)\left[ \hat{Z},[\hat{Z},\overline{\rho }(t)]\right] , \end{aligned}$$

where $d(t)\equiv 4\intop _{t_{0}}^{t}dt'\overline{\omega (t)\omega (t')}$. Assuming an auto-correlation function of the form $\overline{\omega (t)\omega (t')}=\overline{\omega _{0}^{2}}\exp \left( -|t-t'|/T\right)$, where $\overline{\omega _{0}^{2}}\equiv \overline{\omega (t)^{2}}$, we have $d(t)=4\overline{\omega _{0}^{2}}T\left( 1-e^{-(t-t_{0})/T}\right)$ for $t>t_{0}$.

Written in the $\hat{Z}$-eigenbasis $\left\{ |0\rangle ,|1\rangle \right\}$, the master equation becomes a set of linear ODE’s:

49 $$\begin{aligned} \frac{\partial }{\partial t}\overline{\rho }_{00}(t)= & 0, \end{aligned}$$

50 $$\begin{aligned} \frac{\partial }{\partial t}\overline{\rho }_{11}(t)= & 0,\end{aligned}$$

51 $$\begin{aligned} \frac{\partial }{\partial t}\overline{\rho }_{01}(t)= & -d(t)\overline{\rho }_{01}(t),\end{aligned}$$

52 $$\begin{aligned} \frac{\partial }{\partial t}\overline{\rho }_{10}(t)= & -d(t)\overline{\rho }_{10}(t); \end{aligned}$$

the solutions to which are

53 $$\begin{aligned} \overline{\rho }_{00}(t)= & \rho _{00}(t_{0}),\end{aligned}$$

54 $$\begin{aligned} \overline{\rho }_{11}(t)= & \rho _{11}(t_{0}),\end{aligned}$$

55 $$\begin{aligned} \overline{\rho }_{01}(t)= & \rho _{01}(t_{0})\exp \left\{ -4\overline{\omega _{0}^{2}}T^{2}\left( \frac{t-t_{0}}{T}+e^{-(t-t_{0})/T}-1\right) \right\} ,\end{aligned}$$

56 $$\begin{aligned} \overline{\rho }_{10}(t)= & \rho _{10}(t_{0})\exp \left\{ -4\overline{\omega _{0}^{2}}T^{2}\left( \frac{t-t_{0}}{T}+e^{-(t-t_{0})/T}-1\right) \right\} . \end{aligned}$$

As already discussed in the general case of a single Gaussian random process, the energy population remains constant while the coherence decays. This can be understood from a more physical perspective. Quantum coherence depends on the relative phase between the two components $|0\rangle$ and $|1\rangle$. In an individual realization, the relative phase factor is $\exp \left\{ 2i\intop _{t_{0}}^{t}dt'\omega (t')\right\}$. Since $\omega (t)$ is a random process, the quantity $\intop _{t_{0}}^{t}dt'\omega (t')$ becomes increasingly randomized with the passage of time. When the average is taken over an ensemble, these randomly distributed relative phase factors cancel out, thus suppressing the coherence. This suggests that quantum interference is difficult to observe because random fluctuation is ubiquitous.

As has been shown in the more general case Eqs.(43, 44), the 2nd-order master equation gives exact dynamics. When we work out the exact dynamics directly, the result is indeed found to be consistent, though such a direct calculation is more demanding. Clearly, calculational convenience is one advantage of the master equation approach.

The dynamics of a two-level system under a stochastic Hamiltonian, as studied above, models decohering noise prevalent in many realistic quantum systems. For example, superconducting qubits, such as transmons or flux qubits, are sensitive to magnetic flux noise, which introduces stochastic fluctuations in their transition frequencies. Similarly, nitrogen-vacancy centers in diamond are subject to magnetic field fluctuations (e.g. due to nearby spin impurities), resulting in decoherence in their electronic spin states.

### A pair of two-level systems in magnetic field

Next consider an example of two atoms in magnetic field, each atom being a two-level system. A similar model has been studied in^22,23^. The interaction of the spin with the B-field is given by $\hat{H}(t)=\hbar \Omega (t)\left( \hat{Z}^{I}\otimes \hat{I}^{II}+\hat{I}^{I}\otimes \hat{Z}^{II}\right) \equiv \hbar \Omega (t)\hat{Z}_{total}$, where $\hat{Z}^{j}$ is the usual Pauli *z*-operator of the *j*-th atom. Suppose that the frequency $\Omega (t)$, which is proportional to the B-field strength, is a stationary Gaussian random process of zero mean and that its auto-correlation is of a Markovian type $\overline{\Omega (t)\Omega (t')}=\frac{1}{8}\gamma \delta (t-t')$, where the constant $\gamma$ has dimension of inverse-time. Applying Eq. (39), it can be shown that the 2nd-order master equation is

57 $$\begin{aligned} \frac{\partial }{\partial t}\overline{\rho }(t)=-\frac{1}{16}\gamma \left[ \hat{Z}_{total},[\hat{Z}_{total},\overline{\rho }(t)]\right] . \end{aligned}$$

Suppose the system starts in an entangled state between two atoms $|\Psi (t_{0})\rangle =\frac{1}{\sqrt{2}}\left( |01\rangle +|10\rangle \right)$. Since it is an eigenstate of $\hat{Z}_{total}$, the right-hand side of Eq. (57) is identically zero, thus the system does not evolve (except possibly to an unobservable global phase). So the two atoms remain entangled over time. Indeed, notice that $|01\rangle$ and $|10\rangle$ are degenerate eigenstates with the same energy. Thus any arbitrary superposition state of $|01\rangle$ and $|10\rangle$ will remain unchanged over time; in particular, the coherence between them does not decay. Thus, any state in this degenerate subspace is immune to decoherence, making it a good place to store quantum information^18^.

Let us see what happens if the 2-atom system starts in a different entangled state like $|\Psi (t_{0})\rangle =\frac{1}{\sqrt{2}}\left( |00\rangle +|11\rangle \right)$. Writing the master equation in the energy eigenbasis $\left\{ |00\rangle ,|01\rangle ,|10\rangle ,|11\rangle \right\}$, we obtain a set of decoupled linear ODE’s as usual. The solutions are found to be

58 $$\begin{aligned} \overline{\rho }_{00,00}(t)= & \overline{\rho }_{11,11}(t)=\frac{1}{2},\end{aligned}$$

59 $$\begin{aligned} \overline{\rho }_{00,11}(t)= & \overline{\rho }_{11,00}(t)=\frac{1}{2}\exp \left\{ -\gamma (t-t_{0})\right\} , \end{aligned}$$

while the rest of the matrix elements are identically zero. Note that decoherence occurs here, as is expected, since the initial state does not lie in the decoherence-free subspace. Furthermore, as $t\rightarrow \infty$, the coherence is suppressed to zero and $\overline{\rho }(t)\rightarrow \frac{1}{2}|00\rangle \langle 00|+\frac{1}{2}|11\rangle \langle 11|=\frac{1}{2}|0\rangle \langle 0|\otimes |0\rangle \langle 0|+\frac{1}{2}|1\rangle \langle 1|\otimes |1\rangle \langle 1|$. Interestingly, the two atoms become *disentangled*, as there is no quantum correlation between them. In contrast to the general belief that entanglement leads to decoherence, as is widely studied for open quantum systems, here we find that decoherence can result in disentanglement in the case of a closed system.

As we have just seen, a pure state can become a mixed state through decoherence. Such a non-unitary evolution does not conserve the purity $Tr\left( \rho ^{2}\right)$ in general. In fact, decoherence tends to decrease the purity, leading to a statistical mixture at the level of the ensemble average density matrix.

The dynamics of two entangled atoms in a stochastic magnetic field, as studied above, reflects challenges encountered in realistic quantum systems where entangled states are important for quantum information processing. For instance, trapped-ion quantum computers rely on entangled states of atomic qubits for two-qubit gate operations, and stochastically fluctuating ambient magnetic fields (e.g. due to imperfect shielding) can degrade these states through decoherence. Likewise, neutral atoms in optical tweezers, which are used in quantum simulators and also rely on entanglement, are vulnerable to global magnetic field noise that couples to their spin states.

### Heating of a trapped ion

Consider an ion with mass *M* and charge *e* in a harmonic binding potential with characteristic frequency $\omega _{0}$. The ion is driven by a classical electric field *E*(*t*), which is a stationary Gaussian random process of zero mean. It is more convenient to work in the interaction picture, in which the easily solvable, deterministic evolution induced by the harmonic potential is treated separately. The interaction-picture Hamiltonian is given by $\hat{H}(t)=i\hbar \left[ u(t)\hat{a}^{\dagger }-u^{*}(t)\hat{a}\right]$, where $u(t)=ieE(t)e^{i\omega _{0}t}/\sqrt{2M\hbar \omega _{0}}$ and $\hat{a}$ $(\hat{a}^{\dagger })$ being the zero-time annihilation (creation) operator for the harmonic oscillator. (Throughout this section we work in the interaction picture. The subscripts to denote interaction-picture operators are dropped for notational simplicity.) The evolution is exactly solvable and the analytic results are given in^24^.

Let us derive the 2nd-order master equation for this case. Note that it does not fall into the category of a single real Gaussian random process as in Eq. (30). Since $\overline{\hat{H}}=0$, applying Eq. (25), the effective Hamiltonian is found to be

60 $$\begin{aligned} \hat{H}_{eff}= & \frac{1}{2}\intop _{t_{0}}^{t}dt'\left( \overline{u(t)u^{*}(t')}-\overline{u^{*}(t)u(t')}\right) [\hat{a},\hat{a}^{\dagger }]\nonumber \\= & -\frac{e^{2}}{2M\omega _{0}}\intop _{t_{0}}^{t}dt'\overline{E(t)E(t')}\sin \left[ \omega _{0}(t-t')\right] \hat{I}. \end{aligned}$$

In this case, the 2nd-order contribution to the effective Hamiltonian is non-zero, a consequence of the non-commutativity of $\hat{H}(t)$ and $\hat{U}_{1}(t)$. However, since $\hat{H}_{eff}$ is proportional to $\hat{I}$, the unitary part of the equation of motion results only in an unobservable global phase in this case. For more general cases, $\hat{H}_{eff}$ can be different from the identity $\hat{I}$ and can well lead to non-trivial dynamics. Evaluating the remaining terms in Eq. (23) for this example, we find the following master equation:

61 $$\begin{aligned} \begin{aligned} \frac{\partial }{\partial t}\overline{\rho }(t)=&-{\mathscr {C}}(t)\left( \hat{a}^{\dagger }\hat{a}\overline{\rho }(t)+\overline{\rho }(t)\hat{a}^{\dagger }\hat{a}-2\hat{a}\overline{\rho }(t)\hat{a}^{\dagger }\right) \\&-{\mathscr {C}}(t)\left( \hat{a}\hat{a}^{\dagger }\overline{\rho }(t)+\overline{\rho }(t)\hat{a}\hat{a}^{\dagger }-2\hat{a}^{\dagger }\overline{\rho }(t)\hat{a}\right) \\&-e^{2i\omega _{0}t}\left[ {\mathscr {C}}(t)-i{\mathscr {S}}(t)\right] \left( (\hat{a}^{\dagger })^{2}\overline{\rho }(t)+\overline{\rho }(t)(\hat{a}^{\dagger })^{2}-2\hat{a}^{\dagger }\overline{\rho }(t)\hat{a}^{\dagger }\right) \\&-e^{-2i\omega _{0}t}\left[ {\mathscr {C}}(t)+i{\mathscr {S}}(t)\right] \left( \hat{a}^{2}\overline{\rho }(t)+\overline{\rho }(t)\hat{a}^{2}-2\hat{a}\overline{\rho }(t)\hat{a}\right) , \end{aligned} \end{aligned}$$

where ${\mathscr {C}}(t)$ (${\mathscr {S}}(t)$) are proportional to the incomplete cosine (sine) transform of the field correlation function, viz

62 $$\begin{aligned} {\mathscr {C}}(t)\equiv & \frac{e^{2}}{2M\hbar \omega _{0}}\intop _{t_{0}}^{t}dt'\overline{E(t)E(t')}\cos \left[ \omega _{0}(t-t')\right] ,\end{aligned}$$

63 $$\begin{aligned} {\mathscr {S}}(t)\equiv & \frac{e^{2}}{2M\hbar \omega _{0}}\intop _{t_{0}}^{t}dt'\overline{E(t)E(t')}\sin \left[ \omega _{0}(t-t')\right] . \end{aligned}$$

Assuming $\overline{E(t)E(t')}=\overline{E(0)^{2}}\exp \left( -|t-t'|/T\right)$ and setting $t_{0}=0$ for convenience, we find ${\mathscr {C}}(t)=\left( 1/2\tau _{1}\right)$ $\left\{ e^{-t/T}\left[ \omega _{0}T\sin (\omega _{0}t)-\cos (\omega _{0}t)\right] +1\right\}$ and ${\mathscr {S}}(t)=-\left( 1/2\tau _{1}\right) \left\{ e^{-t/T}\left[ \sin (\omega _{0}t)+\omega _{0}T\cos (\omega _{0}t)\right] -\omega _{0}T\right\}$, where $\tau _{1}$ is the heating time defined as $1/\tau _{1}=\left( e^{2}\overline{E(0)^{2}}/M\hbar \omega _{0}\right) \left( T/(1+\omega _{0}^{2}T^{2})\right)$.

Unlike the case of Eq. (30), the 2nd-order master equation does not generate exact dynamics in this case. To get an approximation of the heating from the ground state (i.e. $\rho _{00}(0)=1$) for a short period of time, let us write the master equation in the energy eigenbasis of the harmonic oscillator,

64 $$\begin{aligned} \frac{\partial }{\partial t}\overline{\rho }_{00}(t)=-2{\mathscr {C}}(t)\overline{\rho }_{00}(t)+2{\mathscr {C}}(t)\overline{\rho }_{11}(t) -\sqrt{2}e^{2i\omega _{0}t}\left[ {\mathscr {C}}(t)-i{\mathscr {S}}(t)\right] \overline{\rho }_{02}(t)-\sqrt{2}e^{-2i\omega _{0}t}\left[ {\mathscr {C}}(t)+i{\mathscr {S}}(t)\right] \overline{\rho }_{20}(t). \end{aligned}$$

Since $\overline{\rho }_{11}(t)$, $\overline{\rho }_{02}(t)$ and $\overline{\rho }_{20}(t)$ are all negligibly small for $t\ll T,\,1/\omega _{0}$, we have $\frac{\partial }{\partial t}\overline{\rho }_{00}(t)\cong -2{\mathscr {C}}(t)\overline{\rho }_{00}(t)$ to the lowest order. In the same manner, since the depopulation $1-\overline{\rho }_{00}(t)$ is perturbatively small, we have $\overline{\rho }_{00}(t)\cong 1$ to the lowest order on the right-hand side. Thus an approximate differential equation is obtained as $\frac{\partial }{\partial t}\overline{\rho }_{00}(t)\cong -2{\mathscr {C}}(t)$. Solving this ODE, we find, to lowest order,

65 $$\begin{aligned} 1-\overline{\rho }_{00}(t)\cong 2\intop _{0}^{t}dt'{\mathscr {C}}(t')\cong \frac{e^{2}\overline{E(0)^{2}}}{2M\hbar \omega _{0}}t^{2}, \end{aligned}$$

which holds for short times and agrees with the analytic result in^24^.

**Fig. 1:**
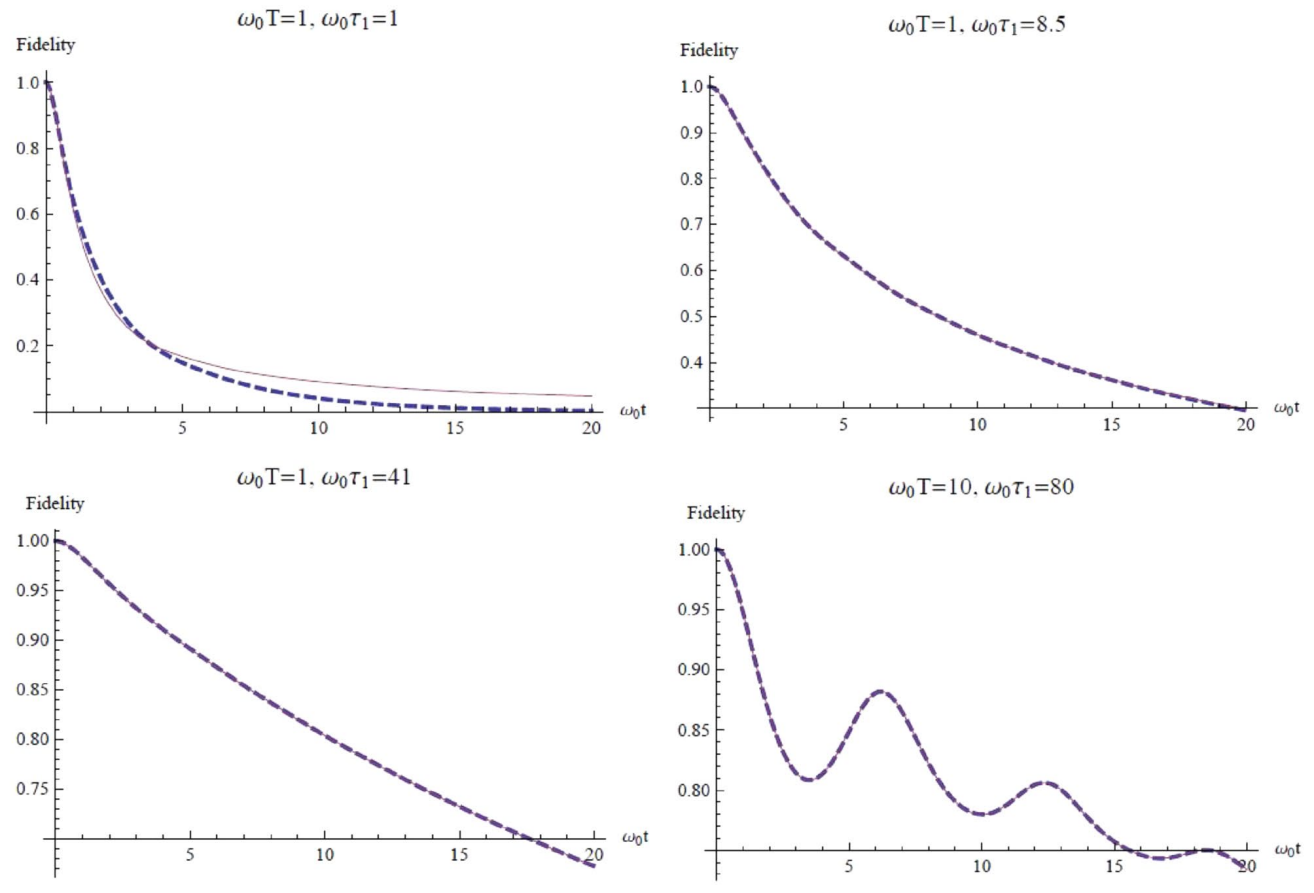
The fidelity of the ground state as a function of dimensionless time $\omega _{0}t$. Dash lines represent our numerical results, while solid lines are exact dynamics from^24^.

To investigate the evolution of the system for longer times, we write down the master equation in the same basis and solve it numerically. Since the Hilbert space is of infinite dimensions, it is not possible to write down the complete set of ODE’s for the matrix elements. Instead, we truncate it to a set of $5\times 5$ coupled ODE’s that includes only the matrix elements of the five lowest energy-eigenstates and their coherence. The numerical solutions of $F(t)\equiv \overline{\rho }_{00}(t)$ (i.e. fidelity of the ground state) are shown in Fig. 1 for different sets of parameters. It can be seen that, as $\omega _{0}\tau _{1}$ (i.e. the dimensionless heating time) increases with $\omega _{0}T$ (i.e. the dimensionless coherence time of $\overline{E(t)E(t')}$) fixed, the numerical result gives better approximation to the exact dynamics. Also note that, for larger values of $\omega _{0}T$, the ground state population shows temporary revival against its general trend of decrease.

Despite the artificial defect caused by the truncation of the set of ODE’s, it is of more interest to know the validity of the 2nd-order master equation itself in approximating the exact dynamics. This is done by comparing the size of higher-order terms to that of the 2nd-order term. Using the Gaussian moment theorem^16^, it is easy to show that ${\mathscr {L}}_{n}[\rho ]=0$ for all odd numbers *n*, so we are interested in the ratios between the even-number-order terms. Assuming $\omega _{0}T$ is fixed, it can be shown that ${\mathscr {L}}_{4}[\rho ]\propto 1/\tau _{1}^{2}\omega _{0}$ as opposed to ${\mathscr {L}}_{2}[\rho ]\propto 1/\tau _{1}$, so ${\mathscr {L}}_{4}[\rho ]/{\mathscr {L}}_{2}[\rho ]\propto 1/\omega _{0}\tau _{1}$. The same ratio holds for ${\mathscr {L}}_{6}[\rho ]/{\mathscr {L}}_{4}[\rho ]$, etc. Therefore, as long as $1/\omega _{0}\tau _{1}$ is small, the higher-order terms become progressively small, lending legitimacy to the 2nd-order approximation. This is also consistent with the previous observation from the numerical results. Physically, this can be better understood by switching to the Schrodinger picture: The external field $H_{field}\propto 1/\tau _{1}$ is treated as a perturbation to the self-Hamiltonian of the system $H_{self}\propto \omega _{0}$. Naturally, as the relative size of the perturbing Hamiltonian $H_{field}/H_{self}\propto 1/\omega _{0}\tau _{1}$ becomes smaller, a perturbative method such as the 2nd-order master equation gives better approximation to the exact dynamics.

## Conclusion

In this paper we have presented the derivation of a formally exact master equation for closed systems driven by stochastic Hamiltonians from an ensemble-average perspective. The principal result of a 2nd-order master equation is given in Eqs.(25, 26). The validity of this approach is examined and the 2nd-order master equation is found to yield either exact dynamics or good approximations to exact dynamics. In particular, we discover that, for the class of problems where the Hamiltonian is proportional to a single Gaussian random process, the 2nd-order master equation is exact.

Applying the formalism to various physical examples, we find the ensemble-average dynamics usually contains decoherence terms in addition to the unitary evolution. Interestingly, we observe that decoherence can lead to disentanglement between two initially entangled atoms in a stochastic magnetic field. Decoherence also plays an important role in the foundational problems of quantum mechanics, as it gives insights in two aspects of the measurement problem, namely the absence of observable superposition and the problem of prefered basis^17^. Extensive research has been done on how environmental entanglement causes decoherence in open systems. However, as our findings suggest, decoherence could also be attributed to the random fluctuations of physical quantities in closed systems. If this is true, then the tension between the classicality of our experience and the quantumness of the underlying laws of physics could be reconciled in some degree by the ubiquitous random fluctuations. Further investigation is needed to find out (a) to what extent decoherence in the natural world is actually caused by random fluctuations and (b) whether/how we can distinguish it from the usual entanglement-induced decoherence through physical observation.

Stochasticity is traditionally viewed as an obstacle in quantum information processing. However, our paper suggests it might also be used as a resource. As we have seen in the physical examples, classical noise can lead to similar effects on the system’s dynamics like system-bath entanglement. Can we then use tailored stochastic Hamiltonians (without system-bath entanglement) to simulate the dynamics of open quantum systems? On the other hand, the noise-induced decoherence-free subspace as identified in the two-atom example could be used to protect quantum information. Further efforts may be made on turning stochasticity into a useful resource.

## Data Availability

The datasets used and/or analysed during the current study are available from the corresponding author on reasonable request.
